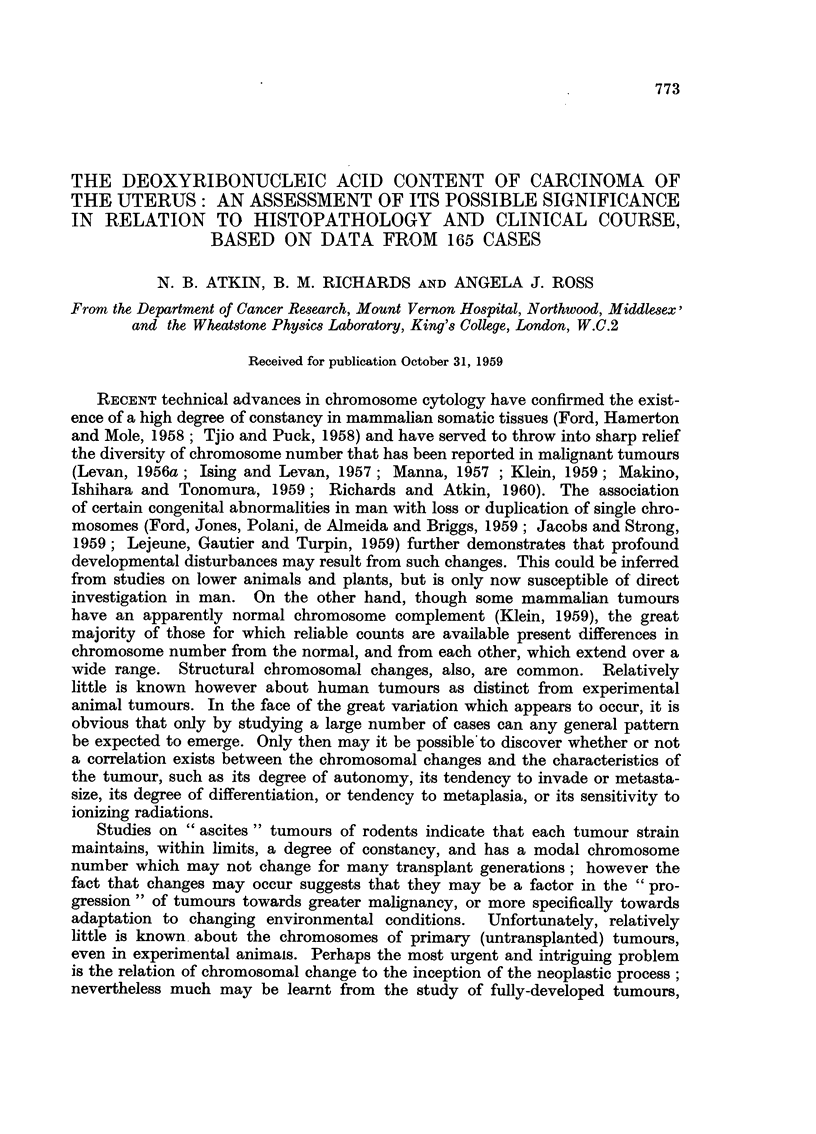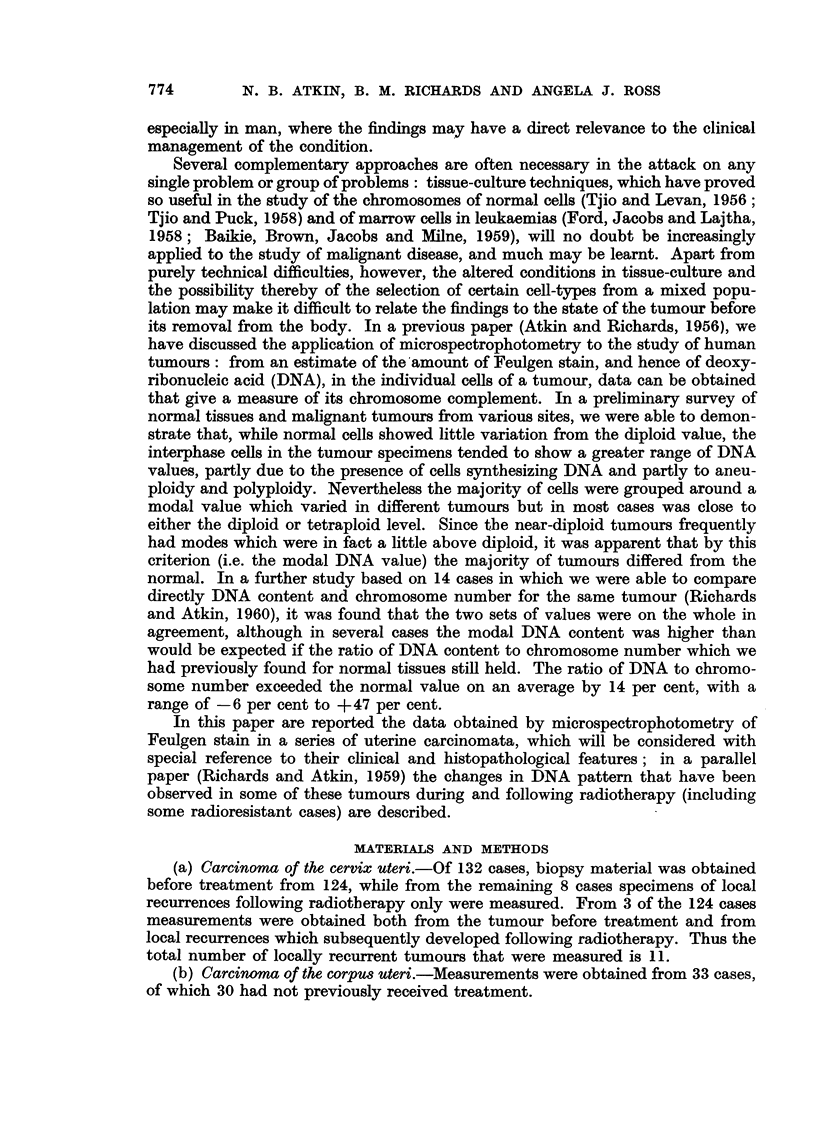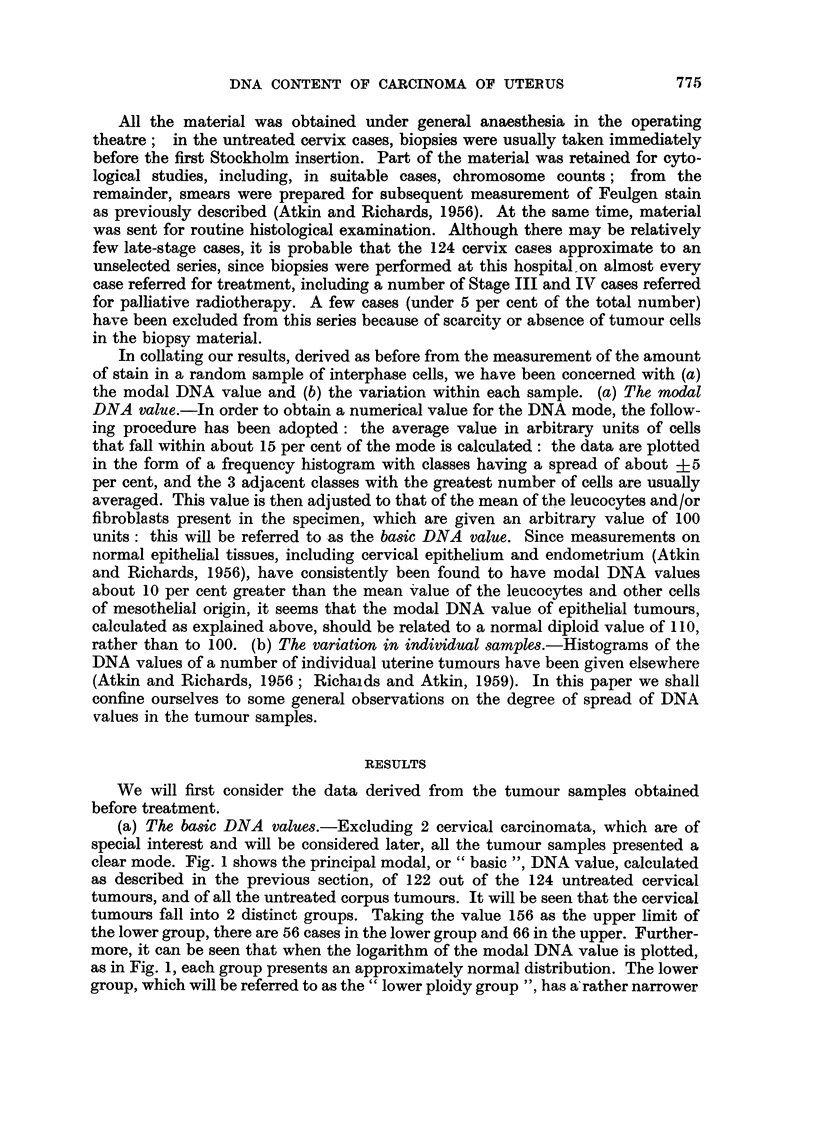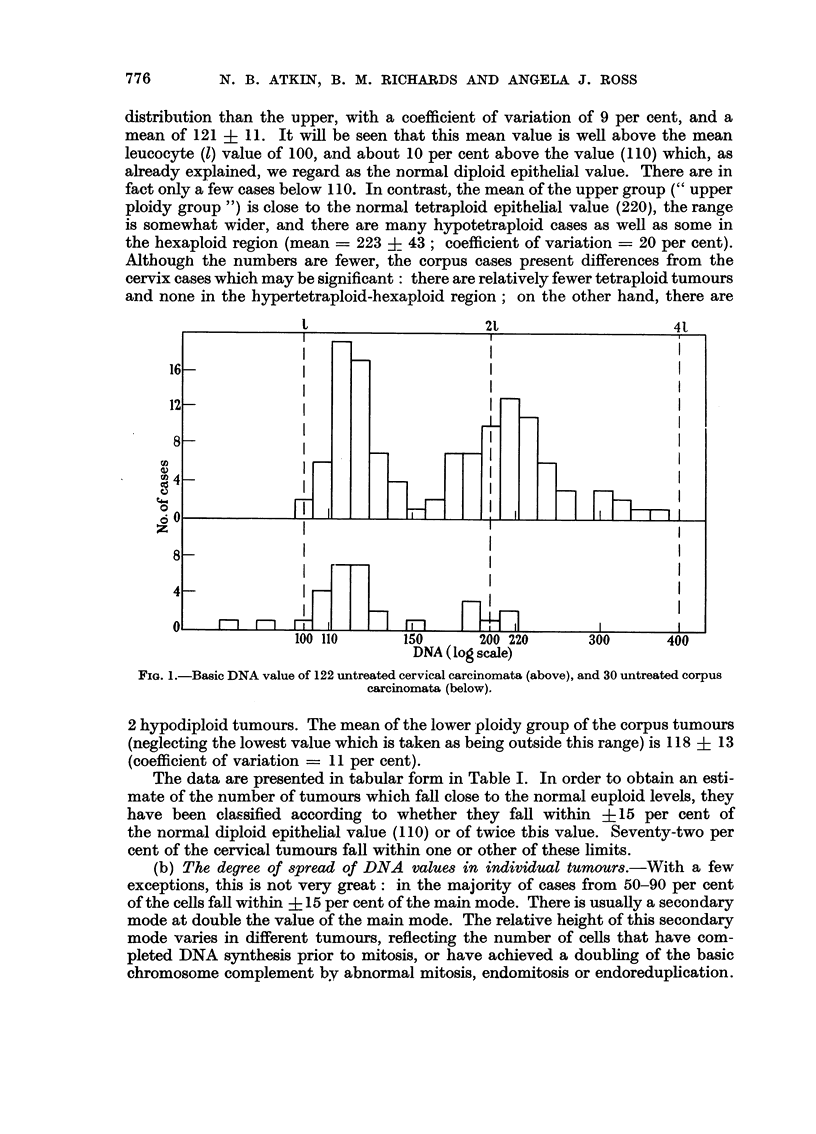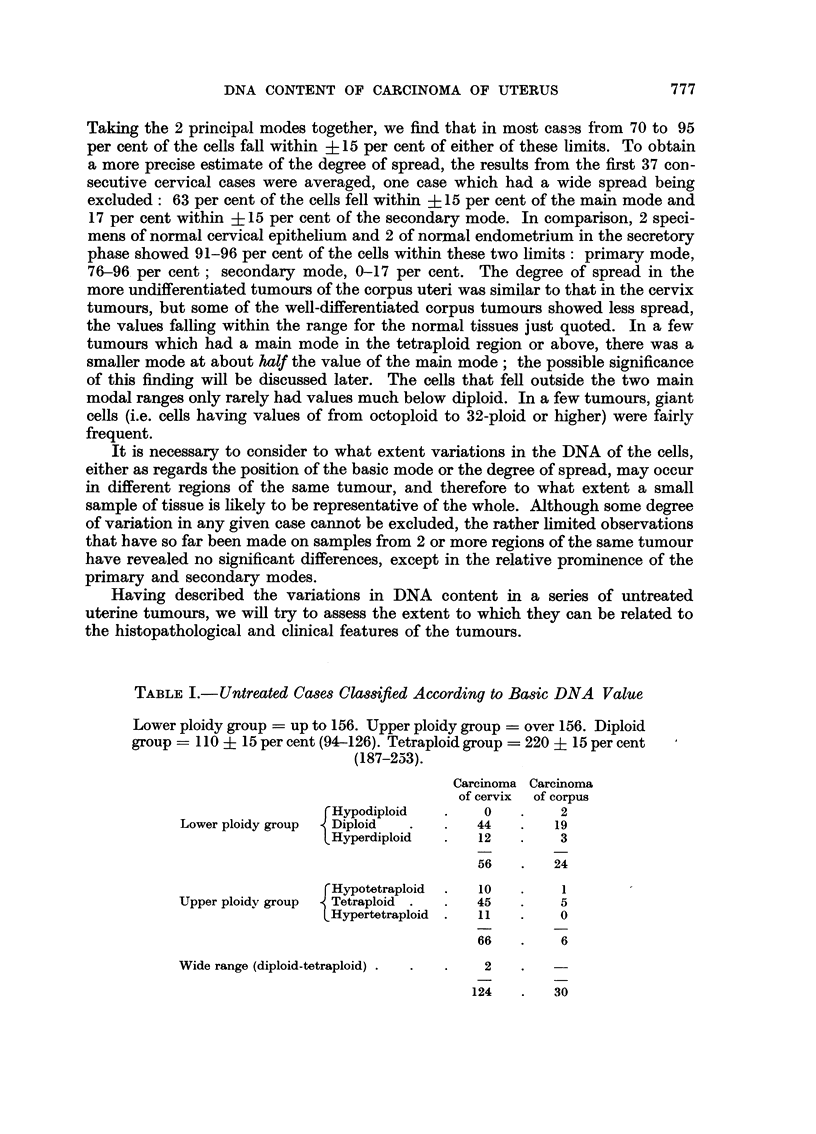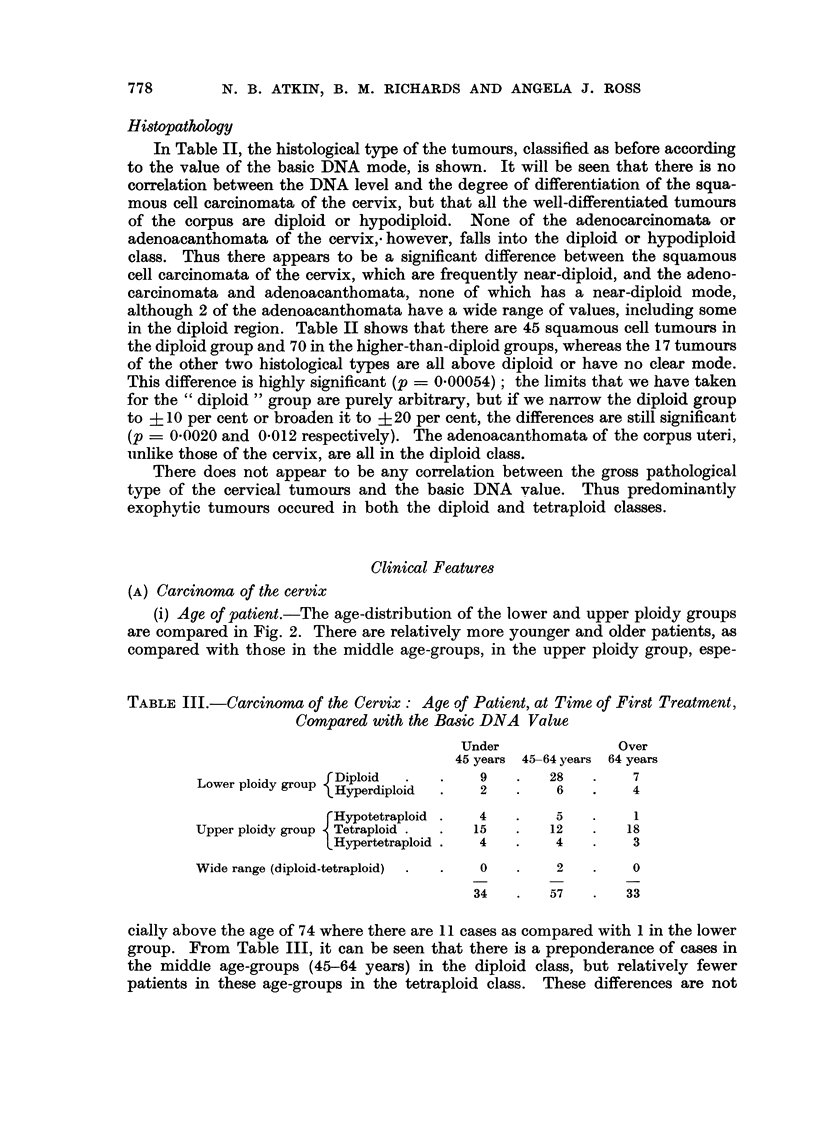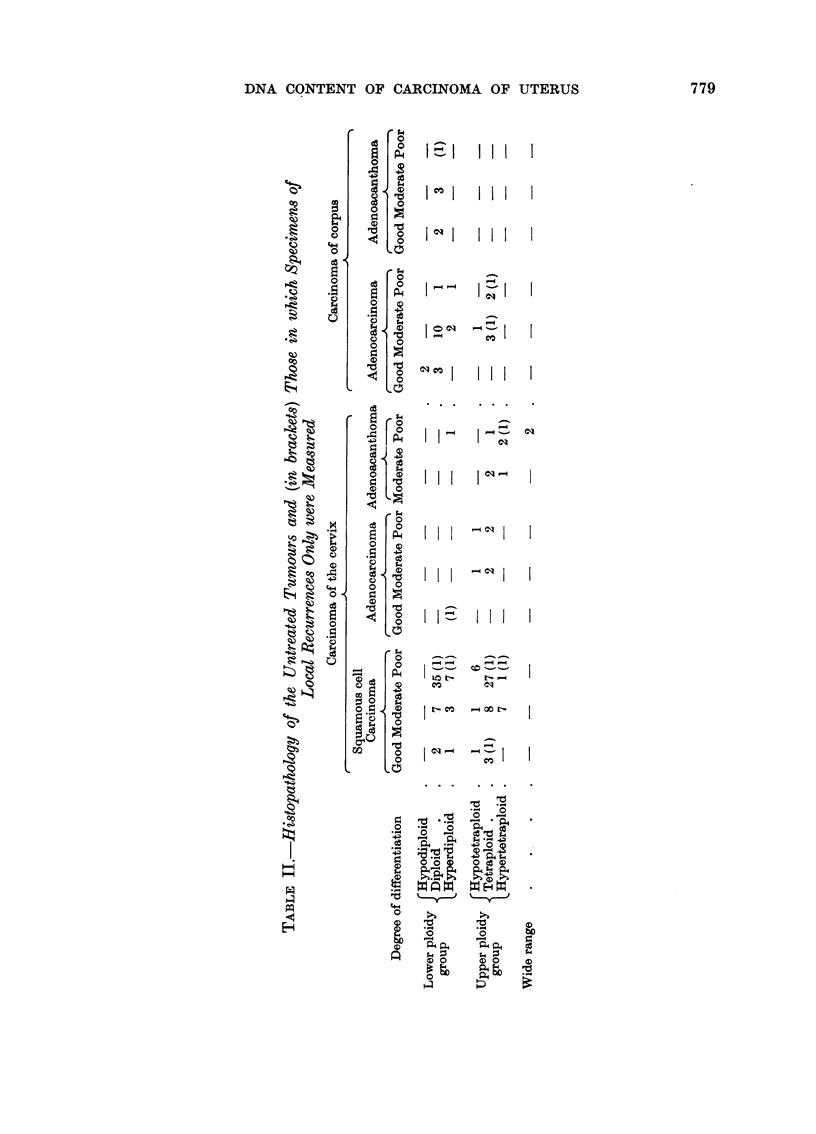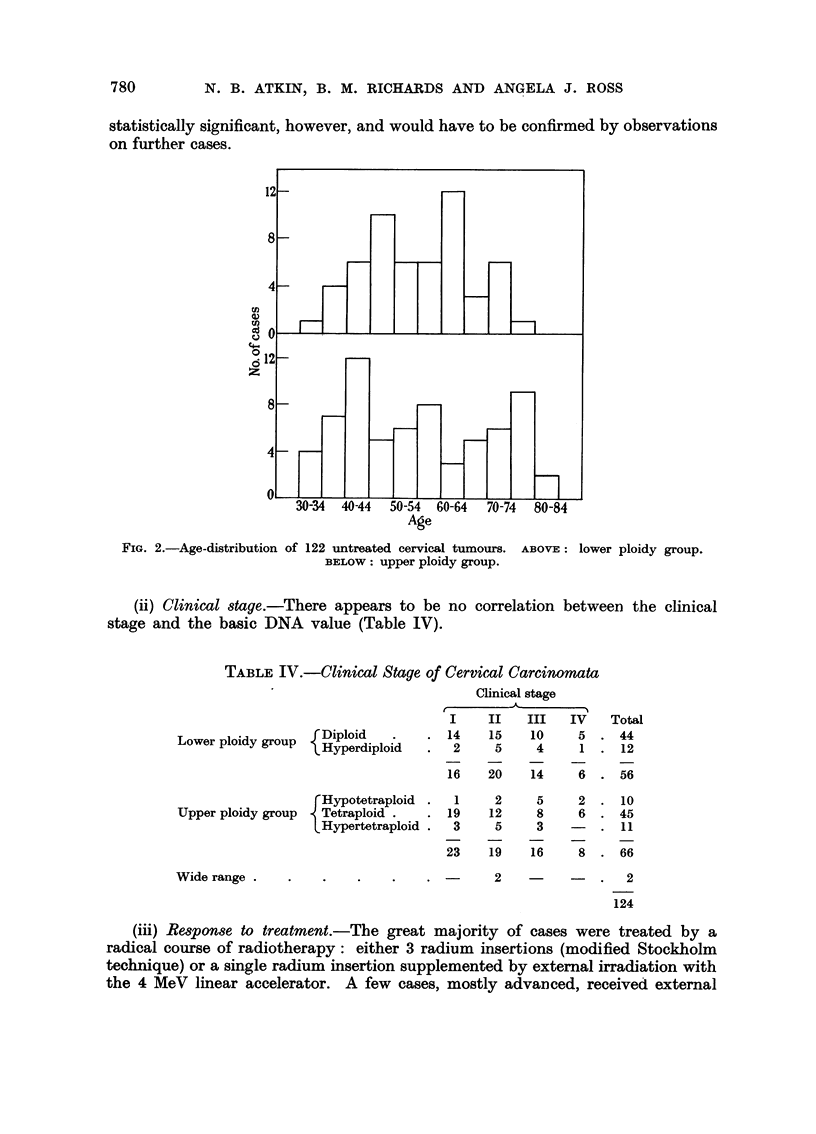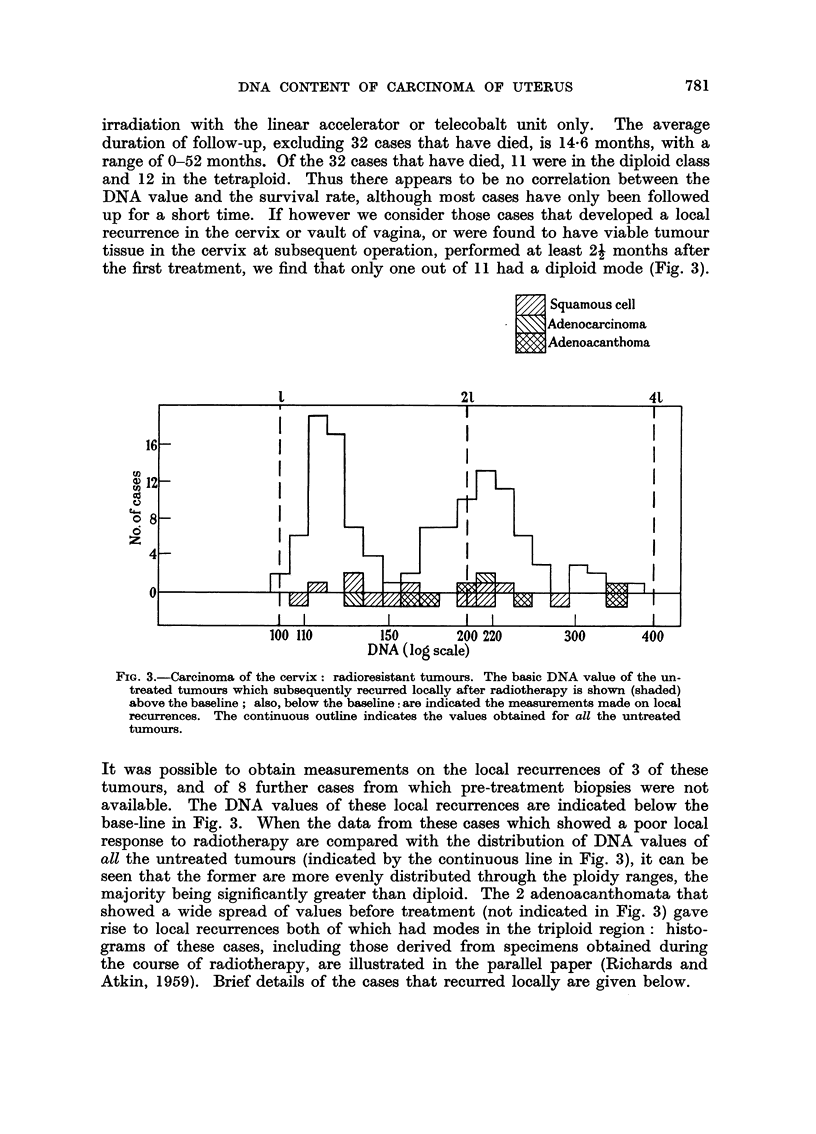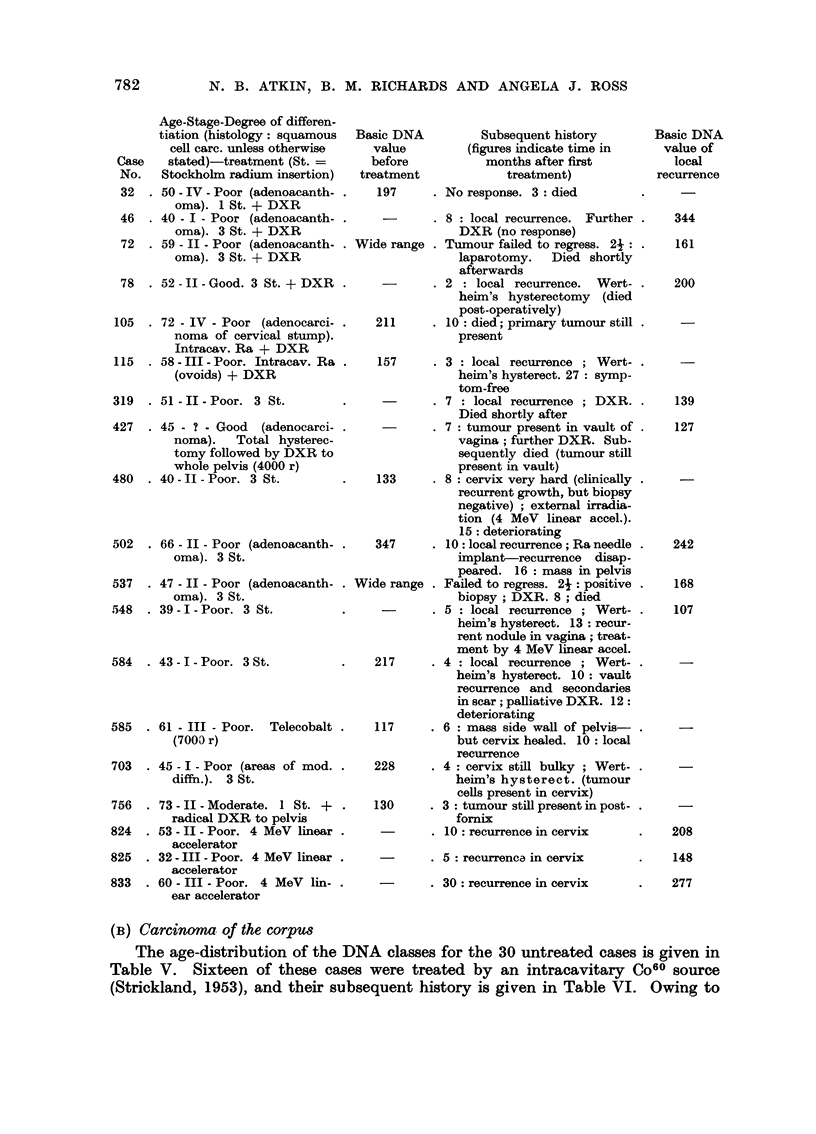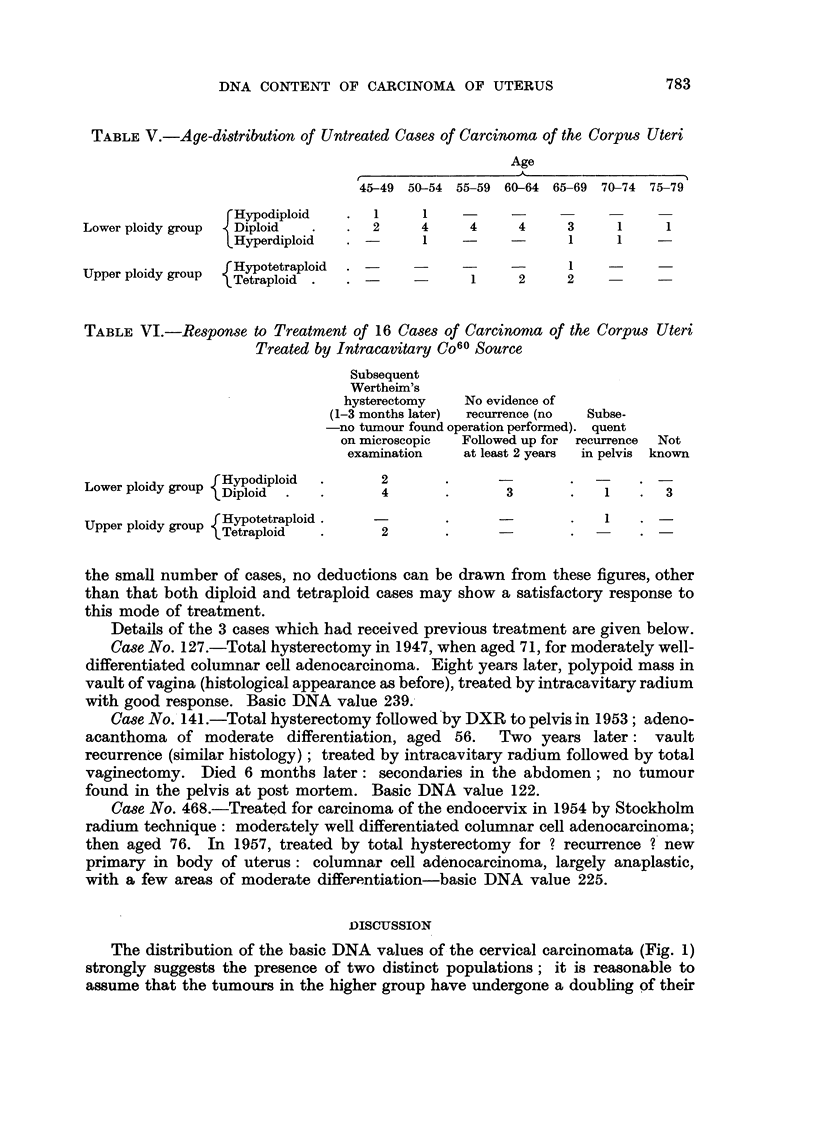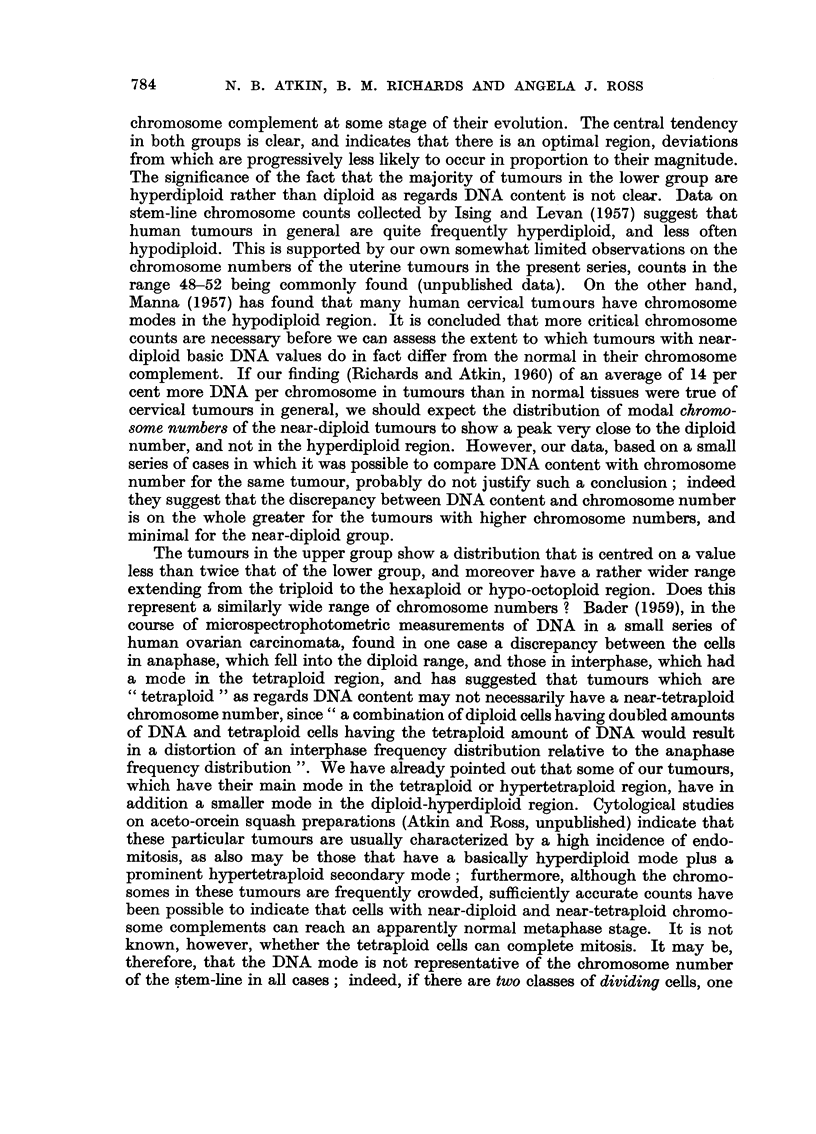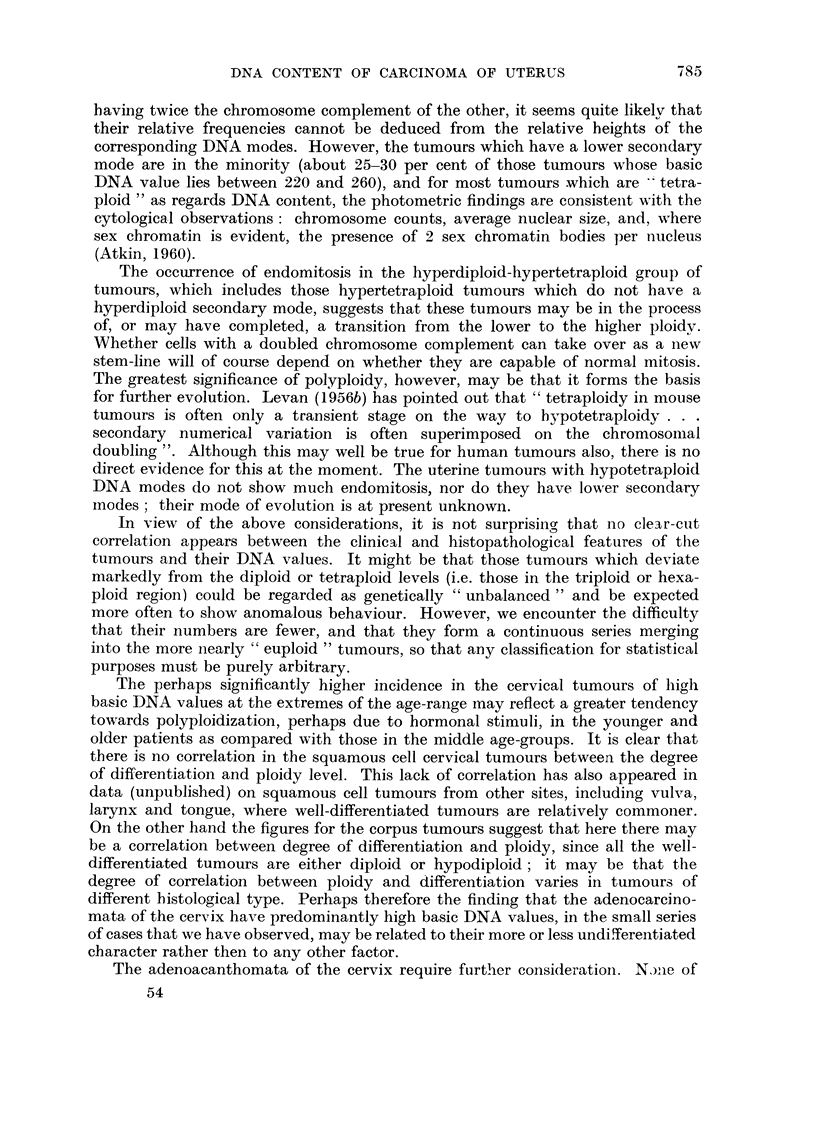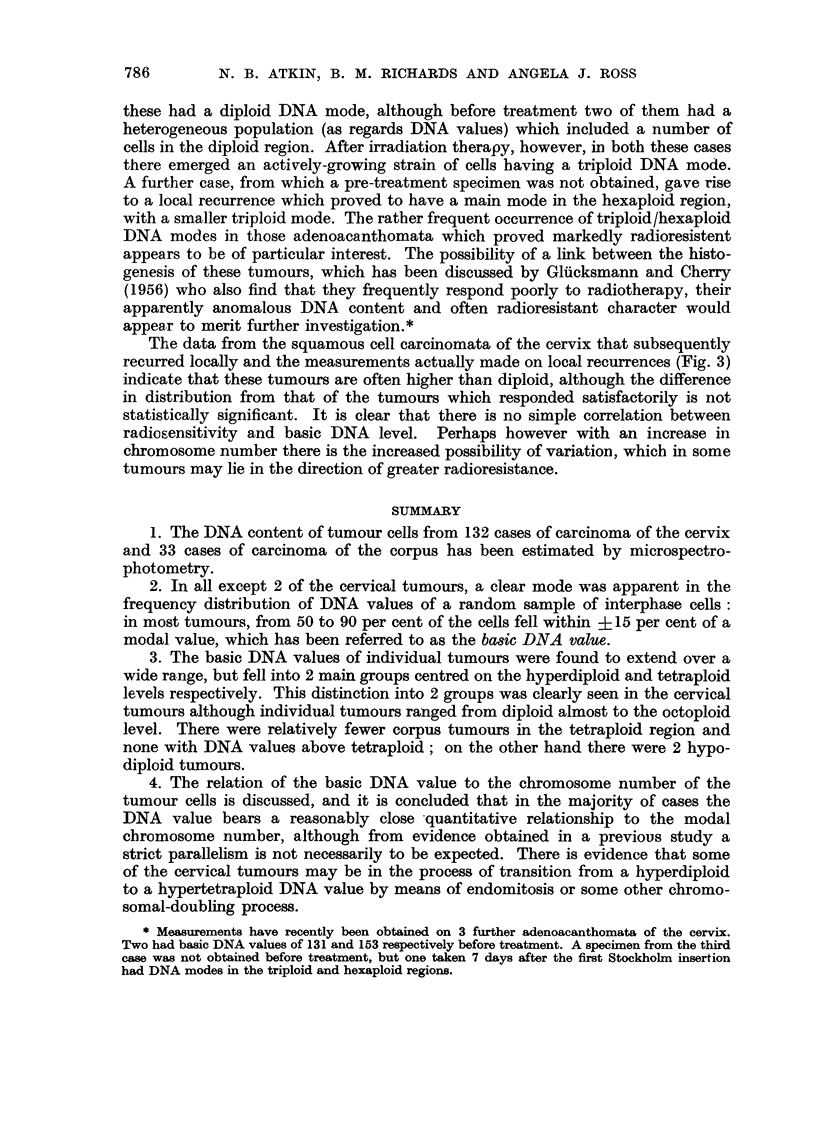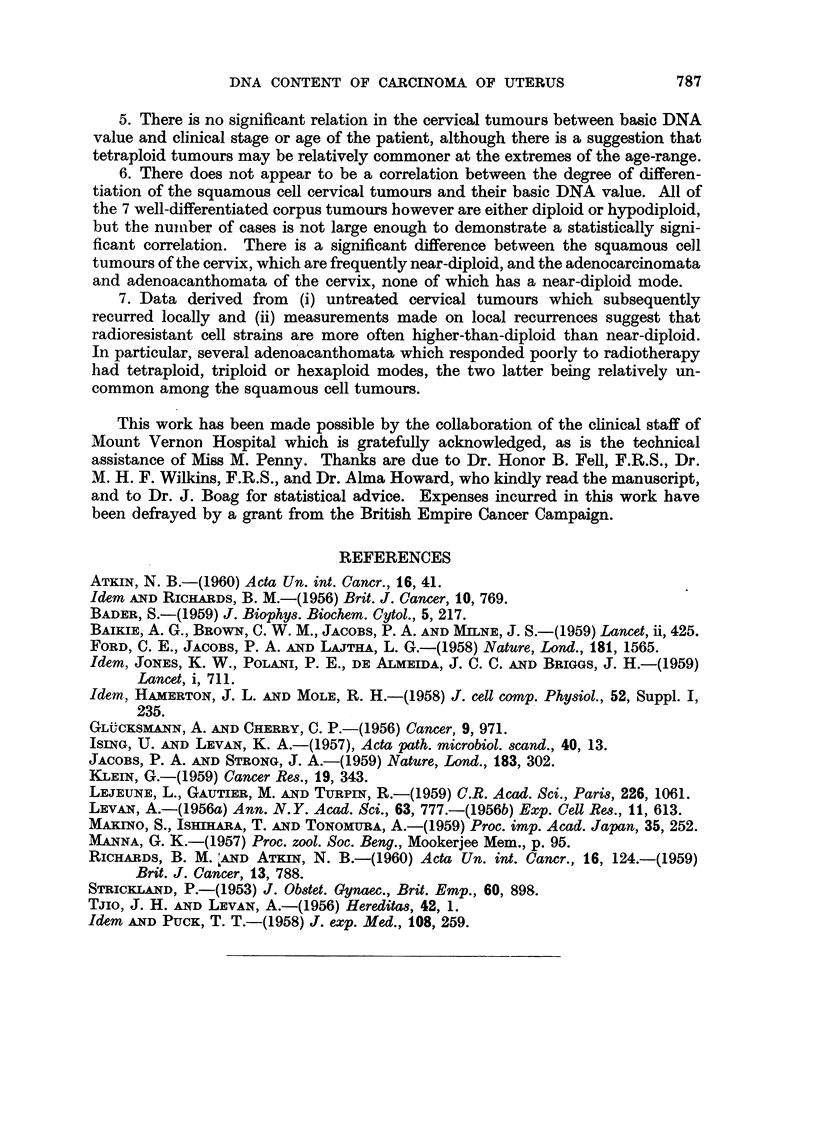# The Deoxyribonucleic Acid Content of Carcinoma of the Uterus: an Assessment of its Possible Significance in Relation to Histopathology and Clinical Course, Based on Data from 165 Cases

**DOI:** 10.1038/bjc.1959.86

**Published:** 1959-12

**Authors:** N. B. Atkin, B. M. Richards, Angela J. Ross


### THE DEOXYRIBONUCLEIC ACID CONTENT OF CARCINOMA OF THE UTERUS: AN ASSESSMENT OF ITS POSSIBLE SIGNIFICANCE IN RELATION TO HISTOPATHOLOGY AND CLINICAL COURSE, BASED ON DATA FROM 165 CASES

N. B. ATKIN, B. M. RICHARDS AND ANGELA J. ROSS

*From the Department of Cancer Research, Mount Vernon Hospital, Northwood, Middlesex' and the Wheatstone Physics Laboratory, King's College, London, W.C.2*

Received for publication October 31, 1959

RECENT technical advances in chromosome cytology have confirmed the existence of a high degree of constancy in mammalian somatic tissues (Ford, Hamerton and Mole, 1958 ; Tjio and Puck, 1958) and have served to throw into sharp relief the diversity of chromosome number that has been reported in malignant tumours (Levan, 1956*a* ; Ising and Levan, 1957 ; Manna, 1957 ; Klein, 1959 ; Makino, Ishihara and Tonomura, 1959 ; Richards and Atkin, 1960). The association of certain congenital abnormalities in man with loss or duplication of single chromosomes (Ford, Jones, Polani, de Almeida and Briggs, 1959 ; Jacobs and Strong, 1959 ; Lejeune, Gautier and Turpin, 1959) further demonstrates that profound developmental disturbances may result from such changes. This could be inferred from studies on lower animals and plants, but is only now susceptible of direct investigation in man. On the other hand, though some mammalian tumours have an apparently normal chromosome complement (Klein, 1959), the great majority of those for which reliable counts are available present differences in chromosome number from the normal, and from each other, which extend over a wide range. Structural chromosomal changes, also, are common. Relatively little is known however about human tumours as distinct from experimental animal tumours. In the face of the great variation which appears to occur, it is obvious that only by studying a large number of cases can any general pattern be expected to emerge. Only then may it be possible to discover whether or not a correlation exists between the chromosomal changes and the characteristics of the tumour, such as its degree of autonomy, its tendency to invade or metastasize, its degree of differentiation, or tendency to metaplasia, or its sensitivity to ionizing radiations.

Studies on " ascites " tumours of rodents indicate that each tumour strain maintains, within limits, a degree of constancy, and has a modal chromosome number which may not change for many transplant generations ; however the fact that changes may occur suggests that they may be a factor in the " progression " of tumours towards greater malignancy, or more specifically towards adaptation to changing environmental conditions. Unfortunately, relatively little is known about the chromosomes of primary (untransplanted) tumours, even in experimental animals. Perhaps the most urgent and intriguing problem is the relation of chromosomal change to the inception of the neoplastic process ; nevertheless much may be learnt from the study of fully-developed tumours, especially in man, where the findings may have a direct relevance to the clinical management of the condition.

Several complementary approaches are often necessary in the attack on any single problem or group of problems : tissue-culture techniques, which have proved so useful in the study of the chromosomes of normal cells (Tjio and Levan, 1956 ; Tjio and Puck, 1958) and of marrow cells in leukaemias (Ford, Jacobs and Lajtha, 1958 ; Baikie, Brown, Jacobs and Milne, 1959), will no doubt be increasingly applied to the study of malignant disease, and much may be learnt. Apart from purely technical difficulties, however, the altered conditions in tissue-culture and the possibility thereby of the selection of certain cell-types from a mixed population may make it difficult to relate the findings to the state of the tumour before its removal from the body. In a previous paper (Atkin and Richards, 1956), we have discussed the application of microspectrophotometry to the study of human tumours : from an estimate of the amount of Feulgen stain, and hence of deoxyribonucleic acid (DNA), in the individual cells of a tumour, data can be obtained that give a measure of its chromosome complement. In a preliminary survey of normal tissues and malignant tumours from various sites, we were able to demonstrate that, while normal cells showed little variation from the diploid value, the interphase cells in the tumour specimens tended to show a greater range of DNA values, partly due to the presence of cells synthesizing DNA and partly to aneuploidy and polyploidy. Nevertheless the majority of cells were grouped around a modal value which varied in different tumours but in most cases was close to either the diploid or tetraploid level. Since the near-diploid tumours frequently had modes which were in fact a little above diploid, it was apparent that by this criterion (i.e. the modal DNA value) the majority of tumours differed from the normal. In a further study based on 14 cases in which we were able to compare directly DNA content and chromosome number for the same tumour (Richards and Atkin, 1960), it was found that the two sets of values were on the whole in agreement, although in several cases the modal DNA content was higher than would be expected if the ratio of DNA content to chromosome number which we had previously found for normal tissues still held. The ratio of DNA to chromosome number exceeded the normal value on an average by 14 per cent, with a range of $-6$ per cent to $+47$ per cent.

In this paper are reported the data obtained by microspectrophotometry of Feulgen stain in a series of uterine carcinomata, which will be considered with special reference to their clinical and histopathological features ; in a parallel paper (Richards and Atkin, 1959) the changes in DNA pattern that have been observed in some of these tumours during and following radiotherapy (including some radioresistant cases) are described.

### MATERIALS AND METHODS

(a) *Carcinoma of the cervix uteri.*—Of 132 cases, biopsy material was obtained before treatment from 124, while from the remaining 8 cases specimens of local recurrences following radiotherapy only were measured. From 3 of the 124 cases measurements were obtained both from the tumour before treatment and from local recurrences which subsequently developed following radiotherapy. Thus the total number of locally recurrent tumours that were measured is 11.

(b) *Carcinoma of the corpus uteri.*—Measurements were obtained from 33 cases, of which 30 had not previously received treatment.

All the material was obtained under general anaesthesia in the operating theatre ; in the untreated cervix cases, biopsies were usually taken immediately before the first Stockholm insertion. Part of the material was retained for cytological studies, including, in suitable cases, chromosome counts ; from the remainder, smears were prepared for subsequent measurement of Feulgen stain as previously described (Atkin and Richards, 1956). At the same time, material was sent for routine histological examination. Although there may be relatively few late-stage cases, it is probable that the 124 cervix cases approximate to an unselected series, since biopsies were performed at this hospital on almost every case referred for treatment, including a number of Stage III and IV cases referred for palliative radiotherapy. A few cases (under 5 per cent of the total number) have been excluded from this series because of scarcity or absence of tumour cells in the biopsy material.

In collating our results, derived as before from the measurement of the amount of stain in a random sample of interphase cells, we have been concerned with (*a*) the modal DNA value and (*b*) the variation within each sample. (*a*) *The modal DNA value.*—In order to obtain a numerical value for the DNA mode, the following procedure has been adopted : the average value in arbitrary units of cells that fall within about 15 per cent of the mode is calculated : the data are plotted in the form of a frequency histogram with classes having a spread of about $\pm 5$ per cent, and the 3 adjacent classes with the greatest number of cells are usually averaged. This value is then adjusted to that of the mean of the leucocytes and/or fibroblasts present in the specimen, which are given an arbitrary value of 100 units : this will be referred to as the *basic DNA value.* Since measurements on normal epithelial tissues, including cervical epithelium and endometrium (Atkin and Richards, 1956), have consistently been found to have modal DNA values about 10 per cent greater than the mean value of the leucocytes and other cells of mesothelial origin, it seems that the modal DNA value of epithelial tumours, calculated as explained above, should be related to a normal diploid value of 110, rather than to 100. (b) *The variation in individual samples.*—Histograms of the DNA values of a number of individual uterine tumours have been given elsewhere (Atkin and Richards, 1956 ; Richaıds and Atkin, 1959). In this paper we shall confine ourselves to some general observations on the degree of spread of DNA values in the tumour samples.

### RESULTS

We will first consider the data derived from the tumour samples obtained before treatment.

(a) *The basic DNA values.*—Excluding 2 cervical carcinomata, which are of special interest and will be considered later, all the tumour samples presented a clear mode. Fig. 1 shows the principal modal, or " basic ", DNA value, calculated as described in the previous section, of 122 out of the 124 untreated cervical tumours, and of all the untreated corpus tumours. It will be seen that the cervical tumours fall into 2 distinct groups. Taking the value 156 as the upper limit of the lower group, there are 56 cases in the lower group and 66 in the upper. Furthermore, it can be seen that when the logarithm of the modal DNA value is plotted, as in Fig. 1, each group presents an approximately normal distribution. The lower group, which will be referred to as the " lower ploidy group ", has a rather narrower distribution than the upper, with a coefficient of variation of 9 per cent, and a mean of 121 $\pm$ 11. It will be seen that this mean value is well above the mean leucocyte ($l$) value of 100, and about 10 per cent above the value (110) which, as already explained, we regard as the normal diploid epithelial value. There are in fact only a few cases below 110. In contrast, the mean of the upper group (" upper ploidy group ") is close to the normal tetraploid epithelial value (220), the range is somewhat wider, and there are many hypotetraploid cases as well as some in the hexaploid region (mean = 223 $\pm$ 43 ; coefficient of variation = 20 per cent). Although the numbers are fewer, the corpus cases present differences from the cervix cases which may be significant : there are relatively fewer tetraploid tumours and none in the hypertetraploid-hexaploid region ; on the other hand, there are

Fig. 1.—Basic DNA value of 122 untreated cervical carcinomata (above), and 30 untreated corpus carcinomata (below).

2 hypodiploid tumours. The mean of the lower ploidy group of the corpus tumours (neglecting the lowest value which is taken as being outside this range) is 118 $\pm$ 13 (coefficient of variation = 11 per cent).

The data are presented in tabular form in Table I. In order to obtain an estimate of the number of tumours which fall close to the normal euploid levels, they have been classified according to whether they fall within $\pm$15 per cent of the normal diploid epithelial value (110) or of twice this value. Seventy-two per cent of the cervical tumours fall within one or other of these limits.

(b) *The degree of spread of DNA values in individual tumours.*—With a few exceptions, this is not very great : in the majority of cases from 50–90 per cent of the cells fall within $\pm$15 per cent of the main mode. There is usually a secondary mode at double the value of the main mode. The relative height of this secondary mode varies in different tumours, reflecting the number of cells that have completed DNA synthesis prior to mitosis, or have achieved a doubling of the basic chromosome complement by abnormal mitosis, endomitosis or endoreduplication.

Taking the 2 principal modes together, we find that in most cases from 70 to 95 per cent of the cells fall within $\pm 15$ per cent of either of these limits. To obtain a more precise estimate of the degree of spread, the results from the first 37 consecutive cervical cases were averaged, one case which had a wide spread being excluded : 63 per cent of the cells fell within $\pm 15$ per cent of the main mode and 17 per cent within $\pm 15$ per cent of the secondary mode. In comparison, 2 specimens of normal cervical epithelium and 2 of normal endometrium in the secretory phase showed 91–96 per cent of the cells within these two limits : primary mode, 76–96 per cent ; secondary mode, 0–17 per cent. The degree of spread in the more undifferentiated tumours of the corpus uteri was similar to that in the cervix tumours, but some of the well-differentiated corpus tumours showed less spread, the values falling within the range for the normal tissues just quoted. In a few tumours which had a main mode in the tetraploid region or above, there was a smaller mode at about *half* the value of the main mode ; the possible significance of this finding will be discussed later. The cells that fell outside the two main modal ranges only rarely had values much below diploid. In a few tumours, giant cells (i.e. cells having values of from octoploid to 32-ploid or higher) were fairly frequent.

It is necessary to consider to what extent variations in the DNA of the cells, either as regards the position of the basic mode or the degree of spread, may occur in different regions of the same tumour, and therefore to what extent a small sample of tissue is likely to be representative of the whole. Although some degree of variation in any given case cannot be excluded, the rather limited observations that have so far been made on samples from 2 or more regions of the same tumour have revealed no significant differences, except in the relative prominence of the primary and secondary modes.

Having described the variations in DNA content in a series of untreated uterine tumours, we will try to assess the extent to which they can be related to the histopathological and clinical features of the tumours.

TABLE I.—*Untreated Cases Classified According to Basic DNA Value*

Lower ploidy group = up to 156. Upper ploidy group = over 156. Diploid group = 110 $\pm$ 15 per cent (94–126). Tetraploid group = 220 $\pm$ 15 per cent (187–253).

| | | Carcinoma of cervix | Carcinoma of corpus |
|---|---|---|---|
| Lower ploidy group | Hypodiploid | 0 | 2 |
| | Diploid | 44 | 19 |
| | Hyperdiploid | 12 | 3 |
| | | 56 | 24 |
| Upper ploidy group | Hypotetraploid | 10 | 1 |
| | Tetraploid | 45 | 5 |
| | Hypertetraploid | 11 | 0 |
| | | 66 | 6 |
| Wide range (diploid-tetraploid) | | 2 | — |
| | | 124 | 30 |

*Histopathology*

In Table II, the histological type of the tumours, classified as before according to the value of the basic DNA mode, is shown. It will be seen that there is no correlation between the DNA level and the degree of differentiation of the squamous cell carcinomata of the cervix, but that all the well-differentiated tumours of the corpus are diploid or hypodiploid. None of the adenocarcinomata or adenoacanthomata of the cervix, however, falls into the diploid or hypodiploid class. Thus there appears to be a significant difference between the squamous cell carcinomata of the cervix, which are frequently near-diploid, and the adenocarcinomata and adenoacanthomata, none of which has a near-diploid mode, although 2 of the adenoacanthomata have a wide range of values, including some in the diploid region. Table II shows that there are 45 squamous cell tumours in the diploid group and 70 in the higher-than-diploid groups, whereas the 17 tumours of the other two histological types are all above diploid or have no clear mode. This difference is highly significant ($p = 0{\cdot}00054$) ; the limits that we have taken for the " diploid " group are purely arbitrary, but if we narrow the diploid group to $\pm 10$ per cent or broaden it to $\pm 20$ per cent, the differences are still significant ($p = 0{\cdot}0020$ and $0{\cdot}012$ respectively). The adenoacanthomata of the corpus uteri, unlike those of the cervix, are all in the diploid class.

There does not appear to be any correlation between the gross pathological type of the cervical tumours and the basic DNA value. Thus predominantly exophytic tumours occured in both the diploid and tetraploid classes.

### *Clinical Features*

(A) *Carcinoma of the cervix*

(i) *Age of patient.*—The age-distribution of the lower and upper ploidy groups are compared in Fig. 2. There are relatively more younger and older patients, as compared with those in the middle age-groups, in the upper ploidy group, especially above the age of 74 where there are 11 cases as compared with 1 in the lower group. From Table III, it can be seen that there is a preponderance of cases in the middle age-groups (45–64 years) in the diploid class, but relatively fewer patients in these age-groups in the tetraploid class. These differences are not

TABLE III.—*Carcinoma of the Cervix : Age of Patient, at Time of First Treatment, Compared with the Basic DNA Value*

| | | Under 45 years | 45–64 years | Over 64 years |
|---|---|---|---|---|
| Lower ploidy group | Diploid | 9 | 28 | 7 |
| | Hyperdiploid | 2 | 6 | 4 |
| Upper ploidy group | Hypotetraploid | 4 | 5 | 1 |
| | Tetraploid | 15 | 12 | 18 |
| | Hypertetraploid | 4 | 4 | 3 |
| Wide range (diploid-tetraploid) | | 0 | 2 | 0 |
| | | 34 | 57 | 33 |

TABLE II.—*Histopathology of the Untreated Tumours and (in brackets) Those in which Specimens of Local Recurrences Only were Measured*

| | | Carcinoma of the cervix | | | | | | | | Carcinoma of corpus | | | | | |
|---|---|---|---|---|---|---|---|---|---|---|---|---|---|---|---|
| | | Squamous cell Carcinoma | | | Adenocarcinoma | | | Adenoacanthoma | | Adenocarcinoma | | | Adenoacanthoma | | |
| Degree of differentiation | | Good | Moderate | Poor | Good | Moderate | Poor | Moderate | Poor | Good | Moderate | Poor | Good | Moderate | Poor |
| Lower ploidy group | Hypodiploid | — | — | — | — | — | — | — | — | 2 | — | — | — | — | — |
| | Diploid | 2 | 7 | 35 (1) | — | — | — | — | — | 3 | 10 | 1 | 2 | 3 | (1) |
| | Hyperdiploid | 1 | 3 | 7 (1) | (1) | — | — | — | 1 | — | 2 | 1 | — | — | — |
| Upper ploidy group | Hypotetraploid | 1 | 1 | 6 | — | 1 | 1 | — | — | — | 1 | — | — | — | — |
| | Tetraploid | 3 (1) | 8 | 27 (1) | — | 2 | 2 | 2 | 1 | — | 3 (1) | 2 (1) | — | — | — |
| | Hypertetraploid | — | 7 | 1 (1) | — | — | — | 1 | 2 (1) | — | — | — | — | — | — |
| Wide range | | — | — | — | — | — | — | — | 2 | — | — | — | — | — | — |

statistically significant, however, and would have to be confirmed by observations on further cases.

FIG. 2.—Age-distribution of 122 untreated cervical tumours. ABOVE : lower ploidy group. BELOW : upper ploidy group.

(ii) *Clinical stage.*—There appears to be no correlation between the clinical stage and the basic DNA value (Table IV).

TABLE IV.—*Clinical Stage of Cervical Carcinomata*

| | | Clinical stage I | II | III | IV | Total |
|---|---|---|---|---|---|---|
| Lower ploidy group | Diploid | 14 | 15 | 10 | 5 | 44 |
| | Hyperdiploid | 2 | 5 | 4 | 1 | 12 |
| | | 16 | 20 | 14 | 6 | 56 |
| Upper ploidy group | Hypotetraploid | 1 | 2 | 5 | 2 | 10 |
| | Tetraploid | 19 | 12 | 8 | 6 | 45 |
| | Hypertetraploid | 3 | 5 | 3 | — | 11 |
| | | 23 | 19 | 16 | 8 | 66 |
| Wide range | | — | 2 | — | — | 2 |
| | | | | | | 124 |

(iii) *Response to treatment.*—The great majority of cases were treated by a radical course of radiotherapy : either 3 radium insertions (modified Stockholm technique) or a single radium insertion supplemented by external irradiation with the 4 MeV linear accelerator. A few cases, mostly advanced, received external irradiation with the linear accelerator or telecobalt unit only. The average duration of follow-up, excluding 32 cases that have died, is 14·6 months, with a range of 0–52 months. Of the 32 cases that have died, 11 were in the diploid class and 12 in the tetraploid. Thus there appears to be no correlation between the DNA value and the survival rate, although most cases have only been followed up for a short time. If however we consider those cases that developed a local recurrence in the cervix or vault of vagina, or were found to have viable tumour tissue in the cervix at subsequent operation, performed at least 2½ months after the first treatment, we find that only one out of 11 had a diploid mode (Fig. 3).

FIG. 3.—Carcinoma of the cervix : radioresistant tumours. The basic DNA value of the untreated tumours which subsequently recurred locally after radiotherapy is shown (shaded) above the baseline ; also, below the baseline ; are indicated the measurements made on local recurrences. The continuous outline indicates the values obtained for *all* the untreated tumours.

It was possible to obtain measurements on the local recurrences of 3 of these tumours, and of 8 further cases from which pre-treatment biopsies were not available. The DNA values of these local recurrences are indicated below the base-line in Fig. 3. When the data from these cases which showed a poor local response to radiotherapy are compared with the distribution of DNA values of *all* the untreated tumours (indicated by the continuous line in Fig. 3), it can be seen that the former are more evenly distributed through the ploidy ranges, the majority being significantly greater than diploid. The 2 adenoacanthomata that showed a wide spread of values before treatment (not indicated in Fig. 3) gave rise to local recurrences both of which had modes in the triploid region : histograms of these cases, including those derived from specimens obtained during the course of radiotherapy, are illustrated in the parallel paper (Richards and Atkin, 1959). Brief details of the cases that recurred locally are given below.

| Case No. | Age-Stage-Degree of differentiation (histology : squamous cell carc. unless otherwise stated)—treatment (St. = Stockholm radium insertion) | Basic DNA value before treatment | Subsequent history (figures indicate time in months after first treatment) | Basic DNA value of local recurrence |
|---|---|---|---|---|
| 32 | 50 - IV - Poor (adenoacanthoma). 1 St. + DXR | 197 | No response. 3 : died | — |
| 46 | 40 - I - Poor (adenoacanthoma). 3 St. + DXR | — | 8 : local recurrence. Further DXR (no response) | 344 |
| 72 | 59 - II - Poor (adenoacanthoma). 3 St. + DXR | Wide range | Tumour failed to regress. 2½ : laparotomy. Died shortly afterwards | 161 |
| 78 | 52 - II - Good. 3 St. + DXR | — | 2 : local recurrence. Wertheim's hysterectomy (died post-operatively) | 200 |
| 105 | 72 - IV - Poor (adenocarcinoma of cervical stump). Intracav. Ra + DXR | 211 | 10 : died ; primary tumour still present | — |
| 115 | 58 - III - Poor. Intracav. Ra (ovoids) + DXR | 157 | 3 : local recurrence ; Wertheim's hysterect. 27 : symptom-free | — |
| 319 | 51 - II - Poor. 3 St. | — | 7 : local recurrence ; DXR. Died shortly after | 139 |
| 427 | 45 - ? - Good (adenocarcinoma). Total hysterectomy followed by DXR to whole pelvis (4000 r) | — | 7 : tumour present in vault of vagina ; further DXR. Subsequently died (tumour still present in vault) | 127 |
| 480 | 40 - II - Poor. 3 St. | 133 | 8 : cervix very hard (clinically recurrent growth, but biopsy negative) ; external irradiation (4 MeV linear accel.). 15 : deteriorating | — |
| 502 | 66 - II - Poor (adenoacanthoma). 3 St. | 347 | 10 : local recurrence ; Ra needle implant—recurrence disappeared. 16 : mass in pelvis | 242 |
| 537 | 47 - II - Poor (adenoacanthoma). 3 St. | Wide range | Failed to regress. 2½ : positive biopsy ; DXR. 8 ; died | 168 |
| 548 | 39 - I - Poor. 3 St. | — | 5 : local recurrence ; Wertheim's hysterect. 13 : recurrent nodule in vagina ; treatment by 4 MeV linear accel. | 107 |
| 584 | 43 - I - Poor. 3 St. | 217 | 4 : local recurrence ; Wertheim's hysterect. 10 : vault recurrence and secondaries in scar ; palliative DXR. 12 : deteriorating | — |
| 585 | 61 - III - Poor. Telecobalt (7000 r) | 117 | 6 : mass side wall of pelvis—but cervix healed. 10 : local recurrence | — |
| 703 | 45 - I - Poor (areas of mod. diffn.). 3 St. | 228 | 4 : cervix still bulky ; Wertheim's hysterect. (tumour cells present in cervix) | — |
| 756 | 73 - II - Moderate. 1 St. + radical DXR to pelvis | 130 | 3 : tumour still present in post-fornix | — |
| 824 | 53 - II - Poor. 4 MeV linear accelerator | — | 10 : recurrence in cervix | 208 |
| 825 | 32 - III - Poor. 4 MeV linear accelerator | — | 5 : recurrence in cervix | 148 |
| 833 | 60 - III - Poor. 4 MeV linear accelerator | — | 30 : recurrence in cervix | 277 |

(B) *Carcinoma of the corpus*

The age-distribution of the DNA classes for the 30 untreated cases is given in Table V. Sixteen of these cases were treated by an intracavitary $Co^{60}$ source (Strickland, 1953), and their subsequent history is given in Table VI. Owing to

TABLE V.—*Age-distribution of Untreated Cases of Carcinoma of the Corpus Uteri*

| | | Age | | | | | | |
|---|---|---|---|---|---|---|---|---|
| | | 45–49 | 50–54 | 55–59 | 60–64 | 65–69 | 70–74 | 75–79 |
| Lower ploidy group | Hypodiploid | 1 | 1 | — | — | — | — | — |
| | Diploid | 2 | 4 | 4 | 4 | 3 | 1 | 1 |
| | Hyperdiploid | — | 1 | — | — | 1 | 1 | — |
| Upper ploidy group | Hypotetraploid | — | — | — | — | 1 | — | — |
| | Tetraploid | — | — | 1 | 2 | 2 | — | — |

TABLE VI.—*Response to Treatment of* 16 *Cases of Carcinoma of the Corpus Uteri Treated by Intracavitary* $Co^{60}$ *Source*

| | | Subsequent Wertheim's hysterectomy (1–3 months later) —no tumour found on microscopic examination | No evidence of recurrence (no operation performed). Followed up for at least 2 years | Subsequent recurrence in pelvis | Not known |
|---|---|---|---|---|---|
| Lower ploidy group | Hypodiploid | 2 | — | — | — |
| | Diploid | 4 | 3 | 1 | 3 |
| Upper ploidy group | Hypotetraploid | — | — | 1 | — |
| | Tetraploid | 2 | — | — | — |

the small number of cases, no deductions can be drawn from these figures, other than that both diploid and tetraploid cases may show a satisfactory response to this mode of treatment.

Details of the 3 cases which had received previous treatment are given below.

*Case No.* 127.—Total hysterectomy in 1947, when aged 71, for moderately well-differentiated columnar cell adenocarcinoma. Eight years later, polypoid mass in vault of vagina (histological appearance as before), treated by intracavitary radium with good response. Basic DNA value 239.

*Case No.* 141.—Total hysterectomy followed by DXR to pelvis in 1953 ; adeno-acanthoma of moderate differentiation, aged 56. Two years later : vault recurrence (similar histology) ; treated by intracavitary radium followed by total vaginectomy. Died 6 months later : secondaries in the abdomen ; no tumour found in the pelvis at post mortem. Basic DNA value 122.

*Case No.* 468.—Treated for carcinoma of the endocervix in 1954 by Stockholm radium technique : moderately well differentiated columnar cell adenocarcinoma; then aged 76. In 1957, treated by total hysterectomy for ? recurrence ? new primary in body of uterus : columnar cell adenocarcinoma, largely anaplastic, with a few areas of moderate differentiation—basic DNA value 225.

### DISCUSSION

The distribution of the basic DNA values of the cervical carcinomata (Fig. 1) strongly suggests the presence of two distinct populations ; it is reasonable to assume that the tumours in the higher group have undergone a doubling of their chromosome complement at some stage of their evolution. The central tendency in both groups is clear, and indicates that there is an optimal region, deviations from which are progressively less likely to occur in proportion to their magnitude. The significance of the fact that the majority of tumours in the lower group are hyperdiploid rather than diploid as regards DNA content is not clear. Data on stem-line chromosome counts collected by Ising and Levan (1957) suggest that human tumours in general are quite frequently hyperdiploid, and less often hypodiploid. This is supported by our own somewhat limited observations on the chromosome numbers of the uterine tumours in the present series, counts in the range 48–52 being commonly found (unpublished data). On the other hand, Manna (1957) has found that many human cervical tumours have chromosome modes in the hypodiploid region. It is concluded that more critical chromosome counts are necessary before we can assess the extent to which tumours with near-diploid basic DNA values do in fact differ from the normal in their chromosome complement. If our finding (Richards and Atkin, 1960) of an average of 14 per cent more DNA per chromosome in tumours than in normal tissues were true of cervical tumours in general, we should expect the distribution of modal *chromosome numbers* of the near-diploid tumours to show a peak very close to the diploid number, and not in the hyperdiploid region. However, our data, based on a small series of cases in which it was possible to compare DNA content with chromosome number for the same tumour, probably do not justify such a conclusion ; indeed they suggest that the discrepancy between DNA content and chromosome number is on the whole greater for the tumours with higher chromosome numbers, and minimal for the near-diploid group.

The tumours in the upper group show a distribution that is centred on a value less than twice that of the lower group, and moreover have a rather wider range extending from the triploid to the hexaploid or hypo-octoploid region. Does this represent a similarly wide range of chromosome numbers ? Bader (1959), in the course of microspectrophotometric measurements of DNA in a small series of human ovarian carcinomata, found in one case a discrepancy between the cells in anaphase, which fell into the diploid range, and those in interphase, which had a mode in the tetraploid region, and has suggested that tumours which are " tetraploid " as regards DNA content may not necessarily have a near-tetraploid chromosome number, since " a combination of diploid cells having doubled amounts of DNA and tetraploid cells having the tetraploid amount of DNA would result in a distortion of an interphase frequency distribution relative to the anaphase frequency distribution ". We have already pointed out that some of our tumours, which have their main mode in the tetraploid or hypertetraploid region, have in addition a smaller mode in the diploid-hyperdiploid region. Cytological studies on aceto-orcein squash preparations (Atkin and Ross, unpublished) indicate that these particular tumours are usually characterized by a high incidence of endomitosis, as also may be those that have a basically hyperdiploid mode plus a prominent hypertetraploid secondary mode ; furthermore, although the chromosomes in these tumours are frequently crowded, sufficiently accurate counts have been possible to indicate that cells with near-diploid and near-tetraploid chromosome complements can reach an apparently normal metaphase stage. It is not known, however, whether the tetraploid cells can complete mitosis. It may be, therefore, that the DNA mode is not representative of the chromosome number of the stem-line in all cases ; indeed, if there are *two* classes of *dividing* cells, one having twice the chromosome complement of the other, it seems quite likely that their relative frequencies cannot be deduced from the relative heights of the corresponding DNA modes. However, the tumours which have a lower secondary mode are in the minority (about 25–30 per cent of those tumours whose basic DNA value lies between 220 and 260), and for most tumours which are " tetraploid " as regards DNA content, the photometric findings are consistent with the cytological observations : chromosome counts, average nuclear size, and, where sex chromatin is evident, the presence of 2 sex chromatin bodies per nucleus (Atkin, 1960).

The occurrence of endomitosis in the hyperdiploid-hypertetraploid group of tumours, which includes those hypertetraploid tumours which do not have a hyperdiploid secondary mode, suggests that these tumours may be in the process of, or may have completed, a transition from the lower to the higher ploidy. Whether cells with a doubled chromosome complement can take over as a new stem-line will of course depend on whether they are capable of normal mitosis. The greatest significance of polyploidy, however, may be that it forms the basis for further evolution. Levan (1956*b*) has pointed out that " tetraploidy in mouse tumours is often only a transient stage on the way to hypotetraploidy . . . secondary numerical variation is often superimposed on the chromosomal doubling ". Although this may well be true for human tumours also, there is no direct evidence for this at the moment. The uterine tumours with hypotetraploid DNA modes do not show much endomitosis, nor do they have lower secondary modes ; their mode of evolution is at present unknown.

In view of the above considerations, it is not surprising that no clear-cut correlation appears between the clinical and histopathological features of the tumours and their DNA values. It might be that those tumours which deviate markedly from the diploid or tetraploid levels (i.e. those in the triploid or hexaploid region) could be regarded as genetically " unbalanced " and be expected more often to show anomalous behaviour. However, we encounter the difficulty that their numbers are fewer, and that they form a continuous series merging into the more nearly " euploid " tumours, so that any classification for statistical purposes must be purely arbitrary.

The perhaps significantly higher incidence in the cervical tumours of high basic DNA values at the extremes of the age-range may reflect a greater tendency towards polyploidization, perhaps due to hormonal stimuli, in the younger and older patients as compared with those in the middle age-groups. It is clear that there is no correlation in the squamous cell cervical tumours between the degree of differentiation and ploidy level. This lack of correlation has also appeared in data (unpublished) on squamous cell tumours from other sites, including vulva, larynx and tongue, where well-differentiated tumours are relatively commoner. On the other hand the figures for the corpus tumours suggest that here there may be a correlation between degree of differentiation and ploidy, since all the well-differentiated tumours are either diploid or hypodiploid ; it may be that the degree of correlation between ploidy and differentiation varies in tumours of different histological type. Perhaps therefore the finding that the adenocarcinomata of the cervix have predominantly high basic DNA values, in the small series of cases that we have observed, may be related to their more or less undifferentiated character rather then to any other factor.

The adenoacanthomata of the cervix require further consideration. None of these had a diploid DNA mode, although before treatment two of them had a heterogeneous population (as regards DNA values) which included a number of cells in the diploid region. After irradiation therapy, however, in both these cases there emerged an actively-growing strain of cells having a triploid DNA mode. A further case, from which a pre-treatment specimen was not obtained, gave rise to a local recurrence which proved to have a main mode in the hexaploid region, with a smaller triploid mode. The rather frequent occurrence of triploid/hexaploid DNA modes in those adenoacanthomata which proved markedly radioresistent appears to be of particular interest. The possibility of a link between the histogenesis of these tumours, which has been discussed by Glücksmann and Cherry (1956) who also find that they frequently respond poorly to radiotherapy, their apparently anomalous DNA content and often radioresistant character would appear to merit further investigation.*

The data from the squamous cell carcinomata of the cervix that subsequently recurred locally and the measurements actually made on local recurrences (Fig. 3) indicate that these tumours are often higher than diploid, although the difference in distribution from that of the tumours which responded satisfactorily is not statistically significant. It is clear that there is no simple correlation between radiosensitivity and basic DNA level. Perhaps however with an increase in chromosome number there is the increased possibility of variation, which in some tumours may lie in the direction of greater radioresistance.

### SUMMARY

1. The DNA content of tumour cells from 132 cases of carcinoma of the cervix and 33 cases of carcinoma of the corpus has been estimated by microspectrophotometry.

2. In all except 2 of the cervical tumours, a clear mode was apparent in the frequency distribution of DNA values of a random sample of interphase cells : in most tumours, from 50 to 90 per cent of the cells fell within $\pm 15$ per cent of a modal value, which has been referred to as the *basic DNA value*.

3. The basic DNA values of individual tumours were found to extend over a wide range, but fell into 2 main groups centred on the hyperdiploid and tetraploid levels respectively. This distinction into 2 groups was clearly seen in the cervical tumours although individual tumours ranged from diploid almost to the octoploid level. There were relatively fewer corpus tumours in the tetraploid region and none with DNA values above tetraploid ; on the other hand there were 2 hypodiploid tumours.

4. The relation of the basic DNA value to the chromosome number of the tumour cells is discussed, and it is concluded that in the majority of cases the DNA value bears a reasonably close quantitative relationship to the modal chromosome number, although from evidence obtained in a previous study a strict parallelism is not necessarily to be expected. There is evidence that some of the cervical tumours may be in the process of transition from a hyperdiploid to a hypertetraploid DNA value by means of endomitosis or some other chromosomal-doubling process.

* Measurements have recently been obtained on 3 further adenoacanthomata of the cervix. Two had basic DNA values of 131 and 153 respectively before treatment. A specimen from the third case was not obtained before treatment, but one taken 7 days after the first Stockholm insertion had DNA modes in the triploid and hexaploid regions.

5. There is no significant relation in the cervical tumours between basic DNA value and clinical stage or age of the patient, although there is a suggestion that tetraploid tumours may be relatively commoner at the extremes of the age-range.

6. There does not appear to be a correlation between the degree of differentiation of the squamous cell cervical tumours and their basic DNA value. All of the 7 well-differentiated corpus tumours however are either diploid or hypodiploid, but the number of cases is not large enough to demonstrate a statistically significant correlation. There is a significant difference between the squamous cell tumours of the cervix, which are frequently near-diploid, and the adenocarcinomata and adenoacanthomata of the cervix, none of which has a near-diploid mode.

7. Data derived from (i) untreated cervical tumours which subsequently recurred locally and (ii) measurements made on local recurrences suggest that radioresistant cell strains are more often higher-than-diploid than near-diploid. In particular, several adenoacanthomata which responded poorly to radiotherapy had tetraploid, triploid or hexaploid modes, the two latter being relatively uncommon among the squamous cell tumours.

This work has been made possible by the collaboration of the clinical staff of Mount Vernon Hospital which is gratefully acknowledged, as is the technical assistance of Miss M. Penny. Thanks are due to Dr. Honor B. Fell, F.R.S., Dr. M. H. F. Wilkins, F.R.S., and Dr. Alma Howard, who kindly read the manuscript, and to Dr. J. Boag for statistical advice. Expenses incurred in this work have been defrayed by a grant from the British Empire Cancer Campaign.

### REFERENCES

ATKIN, N. B.—(1960) *Acta Un. int. Cancr.*, **16**, 41.
*Idem* AND RICHARDS, B. M.—(1956) *Brit. J. Cancer*, **10**, 769.
BADER, S.—(1959) *J. Biophys. Biochem. Cytol.*, **5**, 217.
BAIKIE, A. G., BROWN, C. W. M., JACOBS, P. A. AND MILNE, J. S.—(1959) *Lancet*, ii, 425.
FORD, C. E., JACOBS, P. A. AND LAJTHA, L. G.—(1958) *Nature, Lond.*, **181**, 1565.
*Idem*, JONES, K. W., POLANI, P. E., DE ALMEIDA, J. C. C. AND BRIGGS, J. H.—(1959) *Lancet*, i, 711.
*Idem*, HAMERTON, J. L. AND MOLE, R. H.—(1958) *J. cell comp. Physiol.*, **52**, Suppl. I, 235.
GLÜCKSMANN, A. AND CHERRY, C. P.—(1956) *Cancer*, **9**, 971.
ISING, U. AND LEVAN, K. A.—(1957), *Acta path. microbiol. scand.*, **40**, 13.
JACOBS, P. A. AND STRONG, J. A.—(1959) *Nature, Lond.*, **183**, 302.
KLEIN, G.—(1959) *Cancer Res.*, **19**, 343.
LEJEUNE, L., GAUTIER, M. AND TURPIN, R.—(1959) *C.R. Acad. Sci., Paris*, **226**, 1061.
LEVAN, A.—(1956*a*) *Ann. N.Y. Acad. Sci.*, **63**, 777.—(1956*b*) *Exp. Cell Res.*, **11**, 613.
MAKINO, S., ISHIHARA, T. AND TONOMURA, A.—(1959) *Proc. imp. Acad. Japan*, **35**, 252.
MANNA, G. K.—(1957) *Proc. zool. Soc. Beng.*, Mookerjee Mem., p. 95.
RICHARDS, B. M. AND ATKIN, N. B.—(1960) *Acta Un. int. Cancr.*, **16**, 124.—(1959) *Brit. J. Cancer*, **13**, 788.
STRICKLAND, P.—(1953) *J. Obstet. Gynaec., Brit. Emp.*, **60**, 898.
TJIO, J. H. AND LEVAN, A.—(1956) *Hereditas*, **42**, 1.
*Idem* AND PUCK, T. T.—(1958) *J. exp. Med.*, **108**, 259.